# A Dual-Role of Gu-4 in Suppressing HMGB1 Secretion and Blocking HMGB1 Pro-Inflammatory Activity during Inflammation

**DOI:** 10.1371/journal.pone.0089634

**Published:** 2014-03-06

**Authors:** HuiTing Zhou, XueMei Ji, Yun Wu, Ju Xuan, ZhiLin Qi, Lei Shen, Lei Lan, Qing Li, ZhiMin Yin, ZhongJun Li, ZhiHui Zhao

**Affiliations:** 1 Jiangsu Province Key Laboratory for Molecular and Medicine Biotechnology, College of Life Science, Nanjing Normal University, Nanjing, Jiangsu, PR China; 2 State Key Laboratory of Natural and Biomimetic Drugs, School of Pharmaceutical Sciences, Peking University, Beijing, PR China; 3 Collaborative Innovation Center of Biomedicine for Public Hygiene Emergency and Critical Care, Jiangsu Life Sciences & Technology Innovation Park, Nanjing, Jiangsu, PR China; Southern Medical University, China

## Abstract

**Background:**

High mobility group box 1(HMGB1) was first recognized as a nuclear protein that increased the chromatin remodeling and regulates transcription of many genes. In recent years, HMGB1 has been identified as a critical “late” pro-inflammatory mediator due to its unique secretion pattern and lethal effects in sepsis. Therefore, preventing the active release and inhibiting the pro-inflammatory activity of HMGB1 become promising strategies for the treatment of sepsis. Here, we reported the therapeutic effects of Gu-4, a lactosyl derivative, on sepsis and the underlying molecular mechanisms.

**Methodology/Principal Findings:**

In an experimental rat model of sepsis caused by cecal ligation and puncture (CLP), Gu-4 administration prominently attenuated lung injury and improved the survival of the septic animals, which was positively correlated with the decrease of the serum HMGB1 level. Using RAW264.7 macrophage cell line, we further showed that Gu-4 significantly suppressed the lipopolysaccharide (LPS)-induced release and cytoplasmic translocation of HMGB1. Moreover, Gu-4 not only dose-dependently attenuated recombinant human (rhHMGB1)-induced production of TNF-α, IL-6, and IL-1β in THP-1 cells, but also greatly inhibited the adhesion of rhHMGB1-challenged THP-1 cells to HUVECs. Analyses of flow cytometry demonstrated that Gu-4 could effectively reduce the activation of CD11b elicited by rhHMGB1. Western blot analyses revealed that Gu-4 treatment could partially block the rhHMGB1-induced activation of ERK and NF-κB signalings. Meanwhile, CD11b knockdown also obviously attenuated the rhHMGB1-induced phosphorylations of ERK and IKKα/β.

**Conclusions/Significance:**

Taken together, our results suggest that Gu-4 possesses a therapeutic potential in the treatment of sepsis probably via inhibiting the LPS-induced release of HMGB1 from macrophages and via suppressing the pro-inflammatory activity of HMGB1.

## Introduction

HMGB1 was originally recognized as an intranuclear protein that functions in the maintenance of nucleosome structure, chromatin remodeling, and in the regulation of gene transcription [1]–[2]. In recent years, numerous data from experimental and clinical research highlighted the contributions of extracellular HMGB1 to the pathogenesis of many inflammatory and cancerous diseases such as septic shock [3]–[4]. It is known so far that the high levels of serum HMGB1 under various pathologic states mainly come from two pathways: one is the passive pathway which related with the death and decomposition of cells, the other is the active pathway which connected with non-canonical secretion of HMGB1 from live cells such as macrophages/monocytes when challenged by different stimulators [5]–[7]. The active release pathway of HMGB1 by activated macrophages is dependent on nucleo-cytoplasmic translocation, which is the requirement for HMGB1 extracellular secretion [5], [8].

Once released into extracellular milieu, HMGB1 functions as a potent pro-inflammatory cytokine through activating a wide range of inflammatory responses including massive production of cytokines (e.g., TNF-α, IL-1β, MIP-1, IL-8), expression of adhesion molecules (e.g., ICAM-1, VCAM-1) and chemotactic migration of cells [9]–[12]. HMGB1 mediates cell signaling by binding to the receptors such as RAGE (receptor for advanced glycation end products) [13], TLR-4 (Toll-like receptor) and TLR-2 to activate intracellular signal of mitogen-activated protein kinases (MAPKs) and NF-κB [14]–[15]. The distinct molecular conformations of HMGB1, which are influenced by post-translational modification on the three cysteines (C23, C45, and C106), enable HMGB1 the divergent role in acting as a cytokine-stimulator or as a chemotactic mediator [16]–[18].

Sepsis, a systemic inflammatory responses caused by infection or injury, could lead to the development of tissue damage, septic shock, multiple organ dysfunction syndrome (MODS) and even death [19]. Many therapeutic attempts for sepsis targeting at “early inflammatory mediators” (such as TNF-α, IL-1β, IL-6) came in vain due to the narrow therapeutic window provided by these cytokines [20]–[23]. In recent years, growing evidence has demonstrated that HMGB1 plays a critical role in the generation and development of sepsis by acting as a key “late-phase” mediator [7]. Therefore, for the treatment of sepsis and other diseases, inhibiting HMGB1active release and/or blocking HMGB1 pro-inflammatory activities could be more effective ways to help patients achieve better therapeutic outcomes.

Our previous studies revealed that Gu-4 (N-[2-(1, 3-dilactosyl)-propanyl]-2-amino-pentandiamide), a synthetic oligosaccharide, possessed a therapeutic potential in protecting mice from LPS- or CLP-induced endotoxemia. We further demonstrated that Gu-4 could selectively target CD11b (α subunit of β2 integrin Mac-1) on the surface of leukocytes and inhibit the LPS-induced exposure of CD11b I-domain and the subsequent productions of pro-inflammatory factors to provide protective effects on lethal endotoxemia mice [24]–[25]. However, the detailed molecular mechanisms need to be further investigated. An earlier study by Orlova VV et al. found that CD11b played important roles in the process of leukocyte-endothelial cell adhesion and HMGB1 signaling: the HMGB1-mediated neutrophil recruitment involved a functional interplay between RAGE and Mac-1; HMGB1 prompted the interaction between Mac-1 and RAGE and increased the activity of Mac-1in a RAGE-dependent manner; moreover, HMGB1-induced activation of the transcription factor NF-κB required both the integration of RAGE and Mac-1 on the cell membrane [26]. All these observations led us to speculate that Gu-4 interfering with CD11b could hinder HMGB1 secretion and subsequent HMGB1 signaling.

In this study, we showed that Gu-4 significantly improved the survival of septic animals caused by CLP, which was positively correlated with the decrease of serum HMGB1 level, and further *in vitro* experimental data suggested that Gu-4 exerted its therapeutic effects on CLP-induced sepsis, at least in part, by inhibiting the LPS-induced release of HMGB1 from macrophages and by suppressing the pro-inflammatory activity of HMGB1.

## Materials and Methods

### Antibodies and reagents

LPS (from Escherichia coli 0111: B4), Ethyl Pyruvate (EP), 3- (4, 5-Dimethylthiazol-2-yl)-2,5-diphenyltetrazolium bromide (MTT) and Calcein AM were obtained from Sigma (St.Louis, MO). Recombinant human HMGB1 and anti-HMGB1 monoclonal antibody (mAb) was purchased from R&D Systems (Minneapolis, MN, USA). Phycoerythrin (PE) labeled human CD11b activation-specific antibody (CBRM1/5) was from eBioscience (SanDiego, CA, USA). PE conjugated anti-CD11b antibody (sc-1186) was from Santa Cruz (CA, USA). Anti-human CD11a (Cat. 301214) and anti-human CD11b (Cat. 301312) were from BioLegend (SanDiego, CA). 4, 6-diamidino-2-phenylindole (DAPI) and FITC-conjugated rabbit anti-mouse IgG were obtained from Invitrogen (Carlsbad, CA). Polyclonal antibodies against phosphor-IKKα/β (Ser176/177), IKKβ, NF-κB p65, phospho-IκBα (Ser32), JNK/SAPK, phospho-JNK/SAPK (Thr183/Tyr185), p38 MAPK, phospho-p38 MAPK (Thr180/Tyr182), ERK and phospho-ERK (Thr202/Tyr204) were purchased from Cell Signaling Technology (Beverly, MA, USA). All secondary antibodies used for Western blotting were purchased from Rockland Immunochemical (Gilbertsville, PA).

### Preparation of Gu-4

Lactosyl derivative Gu-4 was designed and synthesized by State Key Laboratory of Natural and Biomimetic of Pharmaceutical Sciences, Peking University [25], [27]–[28]. Endotoxin-free Gu-4 was prepared in our laboratory and confirmed by nuclear magnetic resonance (NMR), mass-spectrometry (MS), elemental analysis and Limulus Amebocyte Lysate assay.

### Cell culture

Murine macrophage-like RAW264.7 cells and THP-1 cells (human acute monocytic leukemia cell line), purchased from the CBCAS (Cell Bank of the Chinese Academic of Sciences, Shanghai, PR China) were cultured in RPMI 1640 medium (Invitrogen) supplemented with 10% fetal bovine serum (Hyclone) and antibiotics (100 U/ml penicillin and 100 µg/ml streptomycin) (Hyclone). Human umbilical vein endothelial cells (HUVECs) obtained from ScienCell (San Diego, CA) were grown in endothelial cell medium (ECM, 1001, ScienCell) with 1% endothelial cell growth supplement (ECGS, 1052, ScienCell). All experiments with HUVECs were performed below 8 passages. All cells were maintained in an atmosphere of humidified 5% CO_2_ at 37°C.

### Animal model of CLP-induced sepsis

Sepsis was induced in male Wistar rats (180–200 g, 7–8 weeks) by cecal ligation and puncture (CLP) as previously described [24], [29]. Rats were anesthetized with sodium pentobarbital (30 mg/kg) before surgery to minimize suffering. In the survival study, sham-treated controls underwent the same surgical procedure (i.e. laparotomy and resuscitation), but the cecum was neither ligated nor punctured. Gu-4 and EP were prepared in solution with 0.9% sterile saline and administered intraperitoneally into rat. Gu-4 (0.929 mg/kg, *i.p.*) was first administrated to the animals 30 min after CLP surgery and was repeated at an interval of 3 h, similar treatment with EP (40 mg/kg, *i.p.*) or saline were taken as controls. Rats were monitored for survival for up to 72 h at a one hour interval. In parallel experiments, blood was collected at the indicated time points after CLP to assay for serum levels of HMGB1 and TNF-α from rats sacrificed under sodium pentobarbital (30 mg/kg, i.p.) anesthesia. After the collection of blood samples, rats were euthanized when they appeared dying (i.e. in a moribund state, as judged by: 1. unresponsive to external stimuli; 2. inability to maintain upright position; 3. agonal breathing). In other parallel experiments, ten hours after CLP, the superior lobe of the right lung was excised from rats sacrificed under sodium pentobarbital (30 mg/kg, i.p.) anesthesia. The *in vivo* study was approved by the Animal Care and Use Committee of Nanjing Normal University and Science and Technology Department of Jiangsu Province [Permit Number: SYXK (Su) 2010-0003]. Animals were handled in accordance with the requirements of Provisions and General Recommendation of Chinese Experimental Animals Administration Legislation.

### Histological examination

Lung tissues isolated from rats were fixed in 10% formalin for 24 h, embedded in paraffin and then serially sectioned. The sections were stained with hematoxylin and eosin (H&E) for histological examination. Lung injuries were evaluated in three parameters including congestion, edema and infiltration of inflammatory cells, to grade the degree of lung injury in 4 fields. Each category was scored according to the following system (0 = normal; 1≤25%; 2 = 25–50%; 3 = 50–75%; 4≥75%) [30] and the mean scores were used to represent the total lung injury.

### Cell viability assay

RAW264.7 cells were seeded into 96-well plates (5×10^4^ cells per well in 150 µL culture medium) and incubated for 12 h. Cells were then treated with different concentrations of Gu-4 or EP in the presence or absence of LPS (100 ng/ml). Three replicates were carried out for each of the different treatments. After 18 h of incubation, cell viability was determined using MTT assay. In brief, 15 µl (5 mg/ml) MTT working solution was pipetted into each well, and the cells were incubated for 4 h with the reagent. Then, after removal of the supernatants without disturbing the crystals in the wells, 200 µl of DMSO was added to each well, and the plates were read using the Synergy2 Multi-Mode Microplate Reader (BIO-TEK, INC.) at 570 nm.

### Cytokine measurement

The amounts of the cytokines in the cell culture supernatants or in the serum of rats including HMGB1, TNF-α, IL-1β and IL-6 were respectively determined by using double-antibody sandwich ELISA kits (HMGB1 kit was purchased from Shanghai Westang Bio-Tech Co, Ltd., Shanghai, China, the other three kits were from R&D Systems), according to the manufacturer's instructions.

### Immunofluorescence microscopy

To detect subcellular localization of HMGB1, RAW264.7 cells were plated on 35-mm glass-bottom dishes and allowed to adhere. In the absence or presence of Gu-4 (80 µM), the cells were stimulated with LPS (100 ng/ml). After 12 h cell samples were fixed in 4% formaldehyde (Sigma) for 20 min at room temperature (RT). Cells were then permeabilized with 0.1% Triton X-100 (Sigma) for 10 min at RT. To reduce non-specific binding cells were blocked in PBS containing 3% bovine serum albumin (BSA) for 1 h at 37°C. Cells were stained with mouse anti-HMGB1 mAb (R&D), followed by a FITC-conjugated secondary antibody (Invitrogen). Finally, cells were incubated with DAPI (4, 6-diamidino-2-phenylindole) for 10 min at RT. Cells were washed three times with PBS between incubation steps above and every time 5 min. Fluorescence of FITC and DAPI were detected by a laser scanning confocal microscopy (Nikon, A1, Japan). The data presented were from one representative experiment of at least 3 independent repeats.

### Adhesion assay

An adhesion assay was performed on HUVECs, according to the procedures described by Yan et al. [24]. HUVECs were seeded in 96-well plates (1×10^5^ cells per well) and cultured to confluence. Aliquots of THP-1 cells (1×10^5^ cells per aliquot) were stained with 8 mM Calcein-AM for 40 min at 37°C, and were washed twice in serum-free medium. The stained THP-1 cells were then co-incubated with 500 ng/ml recombinant HMGB1and different concentrations of Gu-4 or anti-CD11b antibody or anti-CD11a antibody for 20 min at 37°C. The treated THP-1 cells were added to the monolayer of HUVEC in HEPES CaMg buffer (0.05 M HEPES, 0.15 M NaCl, 1 mM CaCl_2_, 1 mM MgCl_2_, pH 7.4) and incubated for 20 min at 37°C. The non-adherent cells were removed by washing with PBS (pH 7.0) and the adherent cells were quantified by fluorescence measurement with a fluorescence plate reader (Bio-Tek SynergyII) at 485 nm/528 nm. The fluorescence intensity is used to represent the number of adherent cells.

### Flow cytometry

Cell surface expression of CD11b and exposure of CD11b active epitope were assessed by flow cytometric assay. Briefly, in the absence (or presence) of Gu-4 (80 µM), THP-1 cells were stimulated with (or without) HMGB1 (200 ng/ml) for 20 min. Then saturating amounts of PE labeled anti-CD11b antibody or PE conjugated CBRM1/5 were added to the cell suspension and incubated for 20 min. The fluorescence of the samples was then immediately measured on a Guava EasyCyte Flow Cytometer (Guava Technologies) and analyzed using the Guava ExpressPro application (Guava CytoSoft Software, version 4.2). Nonspecific fluorescence was determined by using isotype IgG as control.

### Knockdown of CD11b in RAW264.7 cells

RAW 264.7 cells were grown in six-well plates to about 60% confluence. By using Fugene HD Reagent (Roche, Mannheim, Germany), cells were transfected with either a negative control shRNA or SureSilencing shRNA plasmid (GFP) for mouse Itgam (CD11b) according to the protocol from Superarray (SuperArray Bioscience, Frederick, MD, USA). After 48 h transfection, CD11b protein level was determined by Western blotting. The transfected cells were treated with stimulators for the indicated time and subjected to immunoblotting analysis.

### Western blotting

THP-1 cells were rinsed twice with ice-cold PBS, and lysed in lysis buffer containing 20 mM Tris (pH 7.5), 135 mM NaCl, 2 mM ethylenediaminetetraacetic acid (EDTA), 2 mM dithiothreitol (DTT), 25 mM β-glycerophosphate, 2 mM sodium pyrophosphate, 10% glycerol, 1% Triton X-100, 1 mM sodium orthovanadate, 10 mM NaF and 1 mM phenyldulfonyl flouoride (PMSF) supplemented with complete protease inhibitor cocktail (Roche Applied Science, Indianapolis, IN, USA) for 30 min on ice. Nuclear and cytoplasmic extracts were enriched using an NE-PER nuclear and cytoplasmic extraction kit (Pierce Biotechnology Inc., Nepean, Canada). Lysates were centrifuged (15,000× g) at 4°C for 15 min. The Western blot analysis was performed as previously described [31]. The antibody-antigen complexes were visualized by the LI-COR Odyssey Infrared Imaging System (LI-COR Biosciences, Lincoln, NE) using IRDye800 flurophore-conjugated antibody. Quantification on the blot was directly performed using the LI-COR Odyssey Analysis software.

### Statistical analysis

Statistical analysis was performed with Student's t test and one-way ANOVA. Data were presented as means ± SD. Survival of CLP rats was analyzed with Kaplan–Meier survival analysis with the log-rank test for between-group comparisons. Statistical calculations were performed using SPSS of 13.0 version (SPSS Inc., Chicago, IL). A value of p<0.05 was considered significant.

## Results

### Treatment with Gu-4 improved survival of CLP rats and decreased the level of HMGB1 in serum

**Figure 1:**
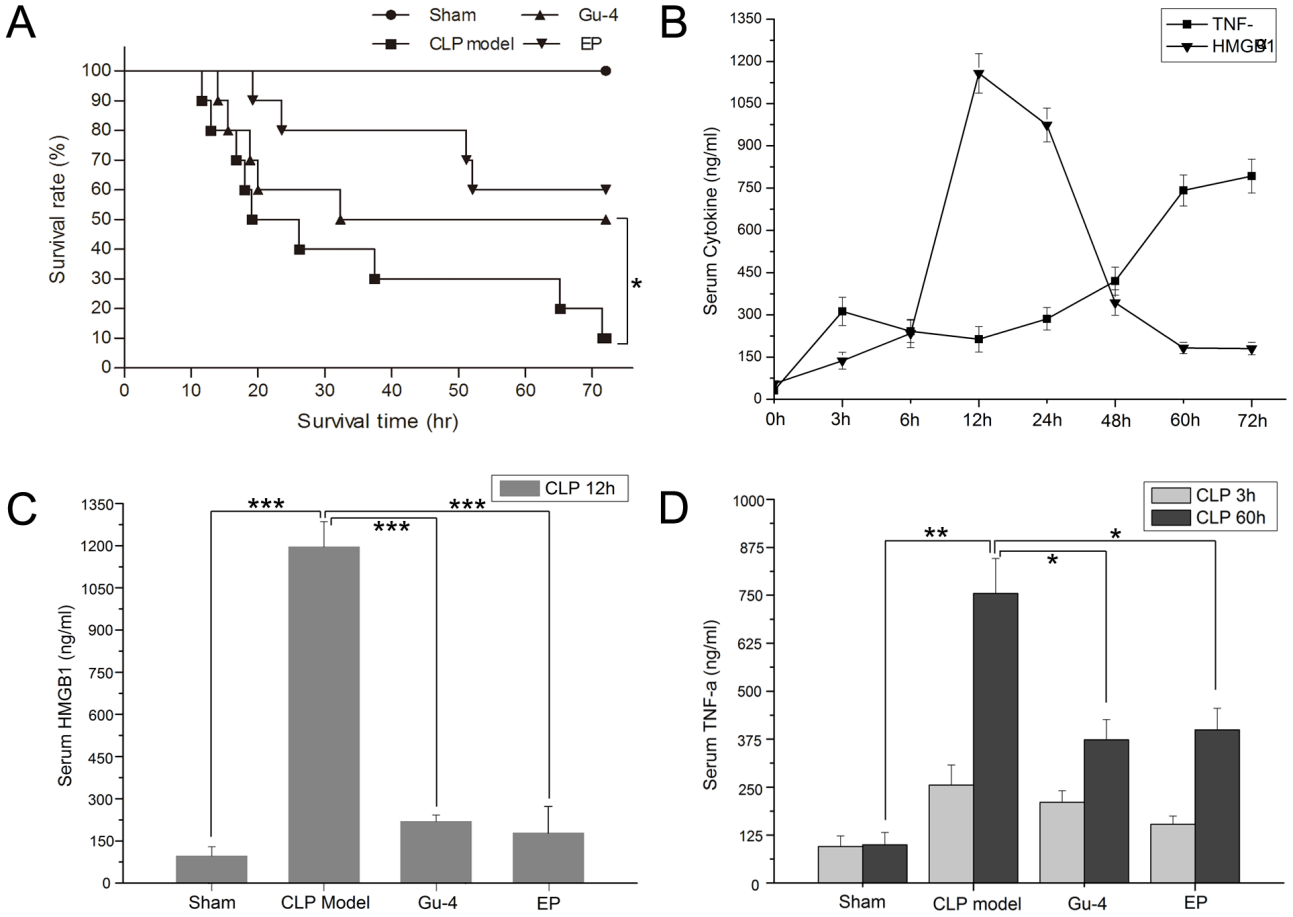
Gu-4 improves survival in septic rats and this improvement is accompanied by marked decrease of HMGB1 and TNF-α level in serum. A: Kaplan-Meier analysis of sepsis lethality confirmed the significant survival benefit in CLP rats with Gu-4 treatment. Gu-4 (0.929 mg/kg, *i.p.*) was first administrated to the animals 30 min after CLP surgery and was repeated at an interval of 3 h, similar treatment with EP (40 mg/kg, *i.p.*) or saline were taken as controls. (n = 10 rats/group) B: The kinetics of serum HMGB1 and TNF-α in a rat model of CLP-induced sepsis. (n = 3 rats/group) C: Gu-4 treatment markedly attenuated HMGB1 release in rats 12 h after CLP. (n = 5 rats/group) D: Gu-4 treatment abolished the second peak of serum TNF-α in rats 60 h after CLP. (n = 5 rats/group) Animals in C and D were undergone the same treatments as those in A. Values are mean ± S.D. *, *p*<0.05; **, *p*<0.01; ***, *p*<0.001.

We previously showed that Gu-4 increased the survival rate but did not affect the expression profiles of serum TNF-α and IL-10 in LPS-induced endotoxemic model. Here, we went forward to evaluate a beneficial effect of Gu-4 on CLP-induced septic animal model and determine the pro-inflammatory cytokines in response to Gu-4 treatment. CLP model is a well established polymicrobial sepsis model, which has been demonstrated to be the most representative animal model of human sepsis [32]. Comparison of survival rates at 72 h after surgery among groups with different treatments clearly showed that administration of Gu-4 (0.929 mg/kg, per 3 h, *i.p.*) significantly improved the survival of rats after CLP (Fig. 1A). In this experiment, we also used Ethyl pyruvate (EP) as a positive control, which has been demonstrated to prevent animals from lethal sepsis and to function as a HMGB1 antagonist [3], [33]–[35]. Both Gu-4 and EP rendered protective effects on septic rats.

As we know, HMGB1, which is released from macrophages, could further stimulate macrophages to produce other inflammatory cytokines like TNF-α to form an inflammatory cascade. Therefore, we detected the dynamic changes of levels of HMGB1 and TNF-α in the sera of CLP rats administrated with saline. As shown in Fig. 1B, the level of serum TNF-α was elevated in a dual-peak manner with two peaks at 3 h and 60 h after CLP surgery, while the level of HMGB1 peaked relatively later at about 12 h after CLP. The second wave of serum TNF-α level was probably induced by further stimulation of the released HMGB1. Next, we compared the levels of HMGB1 and TNF-α in the sera of CLP rats administrated with Gu-4 or EP. As one of HMGB1 antagonist, EP decreased HMGB1 level in CLP-induced serum. Similarly to the effect of EP, Gu-4 significantly inhibited the elevation of HMGB1 at 12 h after CLP (Fig. 1C) and the production of TNF-α at 60 h after CLP, but the early peak of serum TNF-α level was not affected (Fig. 1D).

### Gu-4 alleviated the lung injury in rats after CLP

**Figure 2:**
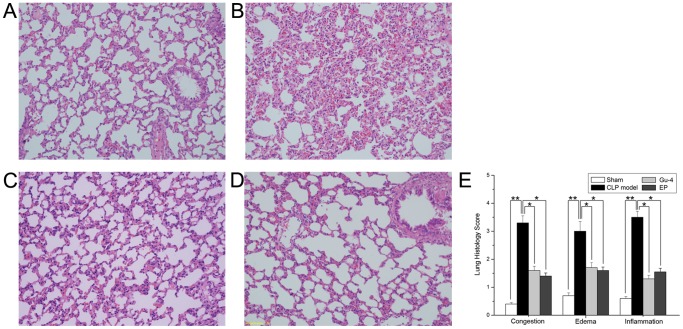
Effects of Gu-4 treatment on lung injury in rats with CLP-induced sepsis. Ten hours after CLP, lung tissues from sham control rats (A), saline treated CLP rats (B), Gu-4 treated CLP rats (C) and EP treated CLP rats (D) were collected and subjected to Hematoxylin and Eosin staining and examined by light microscopy (magnification, ×100). Histological scoring of lung injury were evaluated based on aspects of congestion, edema and inflammation E: The lung injury seriously induced by CLP was greatly attenuated by Gu-4 treatment. Data in E are expressed as mean ± S.D. (n = 5 rat/group) *, *p*<0.05; **, *p*<0.01.

Organ damage is one of leading causes of death in patients with sepsis. In sepsis, lung tissue is particularly susceptible to acute injury. We therefore performed histological examinations and determined the histology scores to evaluate the degree of lung injury characterized by congestion, interstitial edema, and by inflammatory cell infiltration. As shown in Fig. 2, histopathological changes and lung injury were evident in lung tissues from rats with CLP-induced sepsis compared to sham group. It is clear that histological damage and leukocytes infiltration were ameliorated after Gu-4 treatment and Gu-4 group was accompanied with a declined histology score. The positive control EP also attenuated the CLP-induced lung injury.

### Gu-4 inhibited LPS-induced HMGB1 release

**Figure 3:**
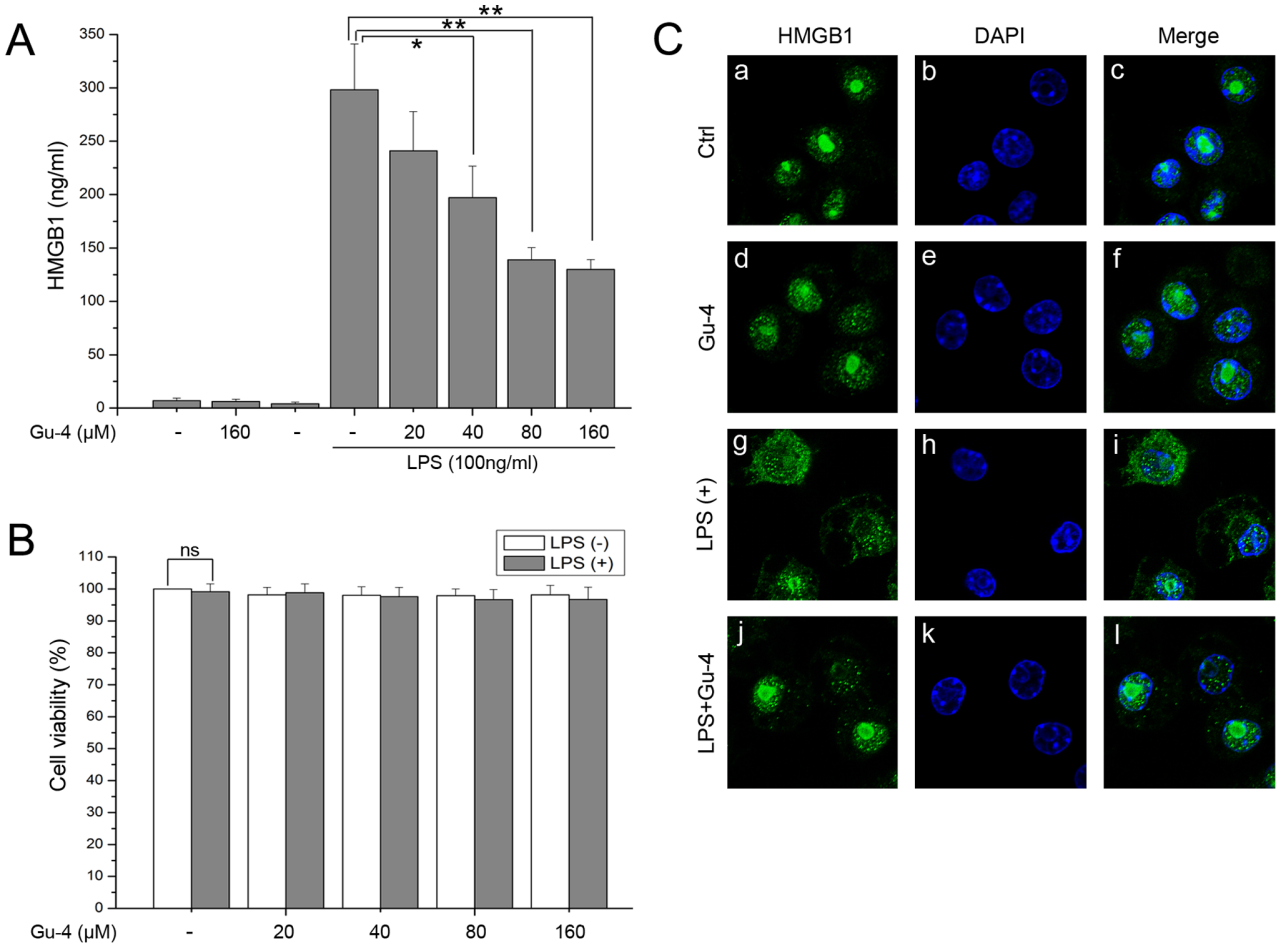
Gu-4 inhibited HMGB1 release and translocation in LPS-stimulated macrophages. A: Gu-4 does-dependently decreases the LPS-induced HMGB1 release from macrophages. RAW264.7 cells were pretreated with the indicated concentrations of Gu-4 for 1 h and then stimulated with LPS (100 ng/ml) for 18 h. Levels of HMGB1 in the culture medium were measured by ELISA. B: In parallel of A, cellular viability evaluation by using MTT assay. The results were expressed as percentage of surviving cells over control group. Data shown are mean ± SD of triplicate experiments. *, *p*<0.05; **, *p*<0.01. ns, non significant. C: Examination of subcellular localization of HMGB1 by confocal microscopy. Under the stimulation of LPS (100 ng/ml) for 12 h, HMGB1 in RAW264.7 cells showed significant re-distribution from to cytoplasm nucleus, while Gu-4 (80 µM) treatment greatly inhibited this LPS elicited intracellular HMGB1 movement. Nucleus (blue, DAPI staining: b, e, h, and k); HMGB1 (green: a, d, g, and j).

To find out whether the decreased serum HMGB1 level in Gu-4 treated CLP animals is due to the inhibitory effect of Gu-4 on HMGB1 release in macrophages, we performed *in vitro* experiments using cultured murine RAW264.7 cells. First, cells were pretreated with Gu-4 (20, 40, 80, 160 µM) for 1 h followed by an 18 h stimulation of LPS (100 ng/ml), and the levels of HMGB1 in the culture medium were determined. As shown in Fig. 3A, LPS alone induced significant HMGB1 release, while pretreatment with Gu-4 attenuated the LPS-induced HMGB1 release in a dose-dependent manner. Cell viability assay showed that there was no statistically significant difference between cells stimulated with or without LPS (Fig. 3B), indicating that LPS-induced HMGB1 release in the medium was mainly come from the active release pathway but not the passive release pathway. And Gu-4 at the tested concentrations did not exhibit cytotoxic effect on the cells treated with or without LPS. We further observed the intracellular distribution of HMGB1 by confocal microscopy. HMGB1 was mainly localized in the nuclei under normal conditions. By contrast, LPS obviously triggered the nucleo-cytoplasmic translocation of HMGB1 as evidenced by the increased staining of HMGB1 in cytoplasm and the decreased staining of HMGB1 in nucleus. The pretreatment with Gu-4 significantly prevented the LPS-induced translocation of HMGB1 (Fig. 3C). These findings suggested that the anti-inflammatory effect of Gu-4 on the sepsis might be involved the suppression of active release of HMGB1 from macrophage cells.

### Gu-4 decreases the production of pro-inflammatory cytokines and adhesion of leukocyte induced by rhHMGB1

**Figure 4:**
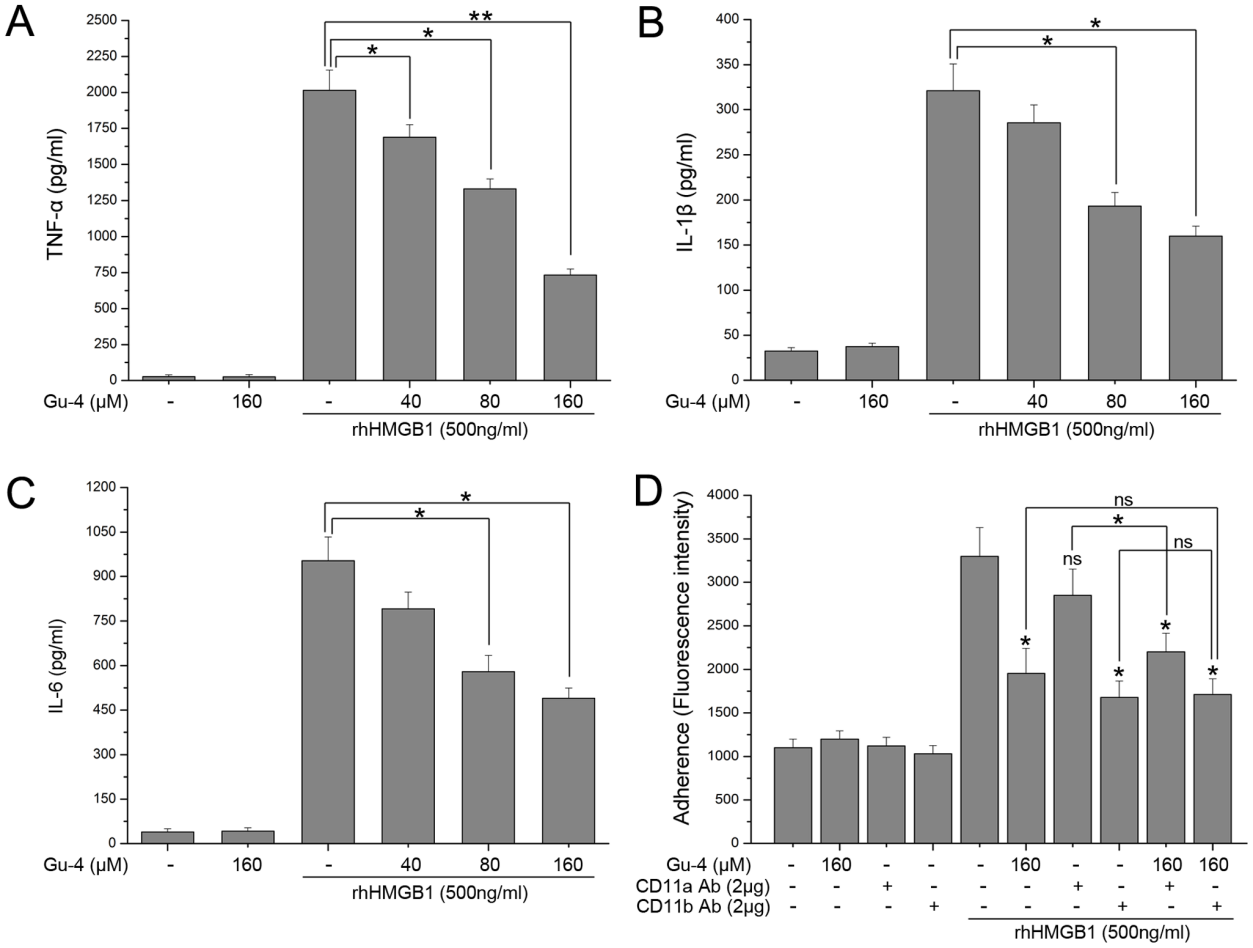
Gu-4 inhibited rhHMGB1-stimulated pro-inflammatory cytokine production and rhHMGB1-induced adhesion of THP-1 cells. A–C: THP-1 cells were pretreated with Gu-4 (0, 40, 80, 160 µM, respectively) for 1 h and then stimulated with rhHMGB1 (500 ng/ml) for 4 h, the levels of TNF-α, IL-6 and IL-1β in the culture medium were measured by ELISA. D: Effects of Gu-4 on rhHMGB1-induced adhesion. Adhesion assay of rhHMGB1-stimulated THP-1 cells with HUVECs was carried out as described in Material and methods. Gu-4 or CD11b antibody, but not CD11a antibody, significantly reduced the adhesion of rhHMGB1-stimulated THP-1 cells to HUVECs. Results are represented as mean ± SD of triplicate experiments. *, *p*<0.05; **, *p*<0.01. ns, non significant.

It has been demonstrated that CD11b on the surface of leukocyte is involved in the pro-inflammatory process of HMGB1 [26], we therefore investigated if Gu-4 could interfere with the CD11b-mediated HMGB1 pro-inflammatory activity. As shown in Fig. 4A–C, when THP-1 cells were stimulated with recombinant human HMGB1 (rhHMGB1, 500 ng/ml) for 4 h, large amounts of pro-inflammatory cytokines including TNF-α, IL-6 and IL-1β were detected in the culture medium, while Gu-4 pretreatment dose-dependently reduced the rhHMGB1-induced production of these cytokines. In addition, we performed adhesion assay to examine the effect of Gu-4 on the rhHMGB1 evoked leukocyte adhesion. As shown in Fig. 4D, rhHMGB1 stimulation dramatically enhanced the adhesion of THP-1 cells to cultured HUVECs, whereas Gu-4 attenuated cell adhesion. Antibody specific against CD11b but not CD11a also showed obvious inhibitory effect on rhHMGB1-triggered cell adhesion. Furthermore, co-treatment of Gu-4 and CD11b blocking antibody did not result in an additive effect, demonstrating that Gu-4 targeted CD11b to suppress HMGB1 pro-inflammatory activity.

### Gu-4 suppresses rhHMGB1-induced activation of CD11b

**Figure 5:**
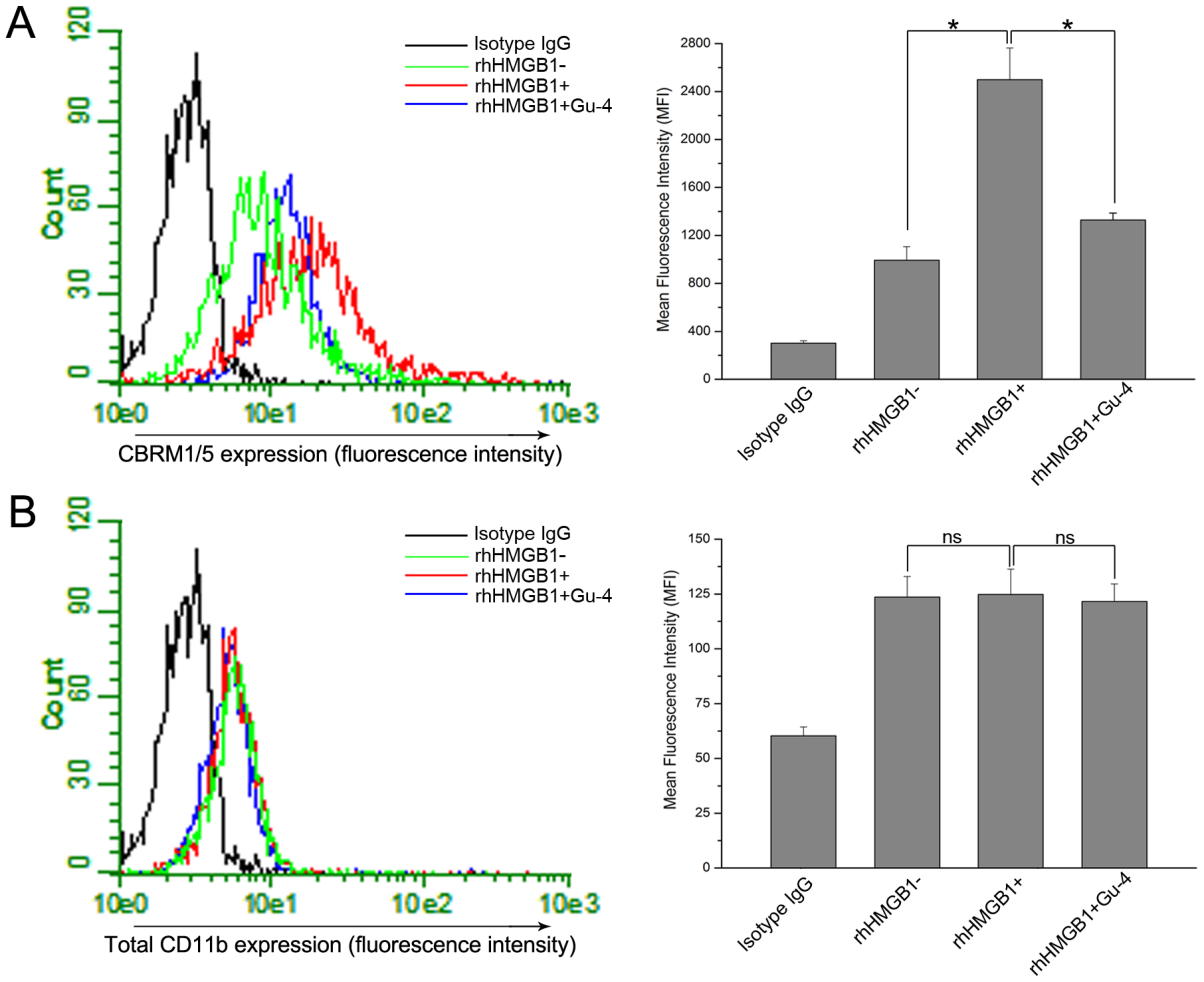
FACS analysis of Gu-4 on the activation and expression of β_2_ integrin CD11b on rhHMGB1-stimulated THP-1 cells. In the absence (or presence) of Gu-4 (80 µM), THP-1 cells were stimulated with (or without) rhHMGB1 (200 ng/ml) for 20 min, then the samples were subjected to co-incubation with saturating amount of PE conjugated CBRM1/5 (or PE labeled sc-1186) for 10 min. Quantitative cell surface expression of CD11b I-domain and CD11b were analyzed by FACS. A: Effects of Gu-4 on the exposure of CD11b I-domain induced by rhHMGB1. B: Effects of Gu-4 on the expression of CD11b induced by rhHMGB1. Data shown are representative results of triplicate experiments. The relative amount was determined by measurement of fluorescence intensity. *, *p*<0.05. ns, non significant.

It is prerequisite that CD11b exposes its I-domain, which is the ligand binding site or active epitope of CD11b, during the process of leukocyte adhesion. We then investigated the effect of Gu-4 on the rhHMGB1-induced activation of CD11b. Flow cytometry was used to determine the activation and the expression of CD11b on cell surface (Fig. 5). Upon rhHMGB1 stimulation, the expression of CD11b active epitope as seen as the fluorescence of CBRM1/5, a PE-conjugated antibody targeting CD11b I-domain, on THP-1 cells was greatly increased compared with that of on the resting cells (Fig. 5A), while the total amount of CD11b on the cell surface was substantially unchanged by this short-term stimulation of rhHMGB1 (Fig. 5B). Treatment with Gu-4 reduced the expression of CD11b active epitope in the presence of rhHMGB1without altering the expression level of CD11b.

### Gu-4 inhibits rhHMGB1-elevated ERK and NF-κB signaling

**Figure 6:**
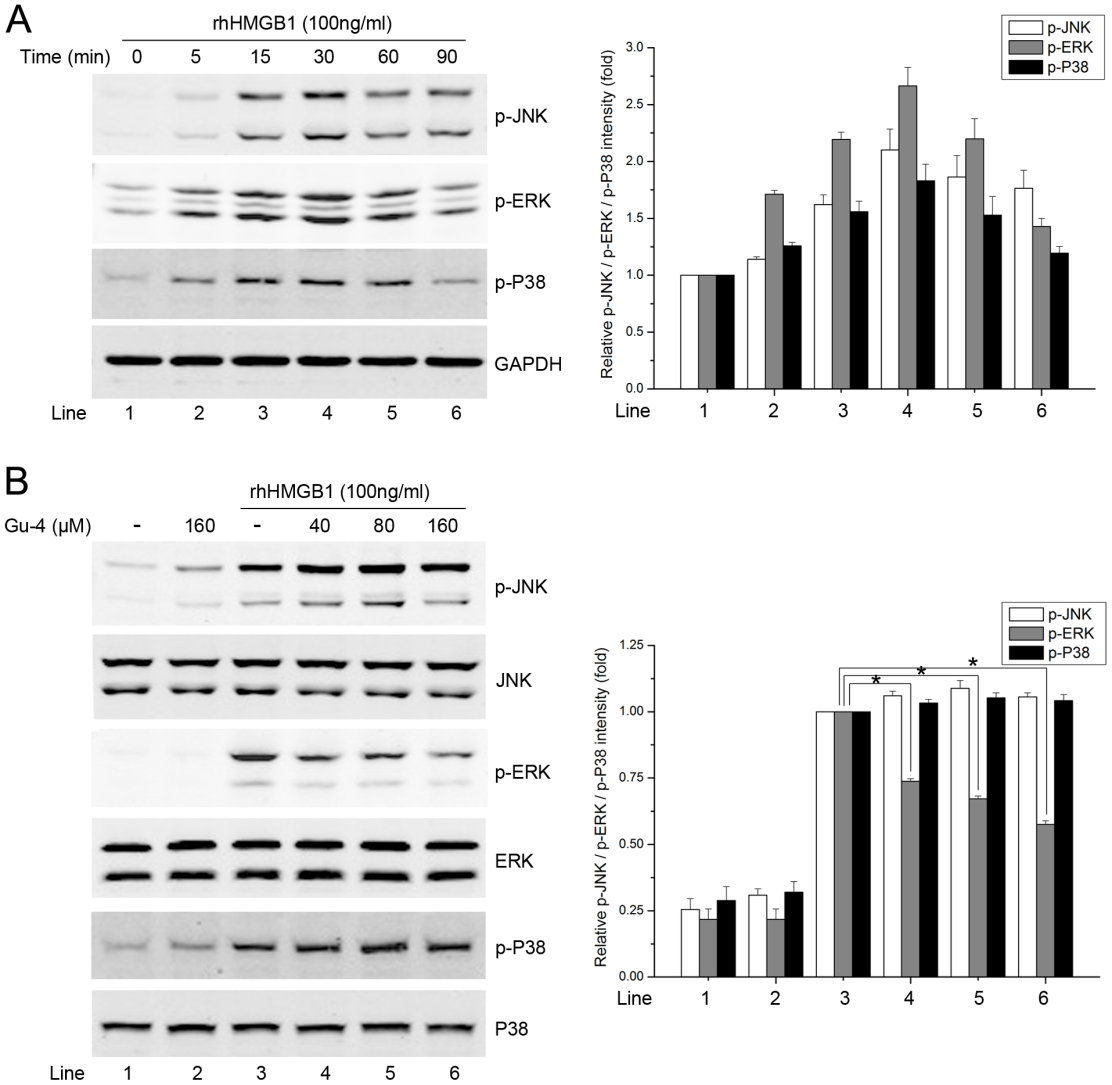
Effects of Gu-4 on rhHMGB1-induced activation of MAPKs. A: Western blot (WB) analysis of the activation of MAPKs including JNK54/46, ERK1/2 and P38 in THP-1 cells treated with rhHMGB1 (100 ng/ml) for 0, 5, 15, 30, 60 or 90 min, respectively. B: THP-1 cells were pretreated with or without Gu-4 (40, 80, 160 µM) for 1 h, followed by stimulating with rhHMGB1 (100 ng/ml) for 30 min. Cell lysate were prepared and the levels of phospho-MAPKs were determined by WB analysis. Data shown are representative results of triplicate experiments. *, *p*<0.05.

**Figure 7:**
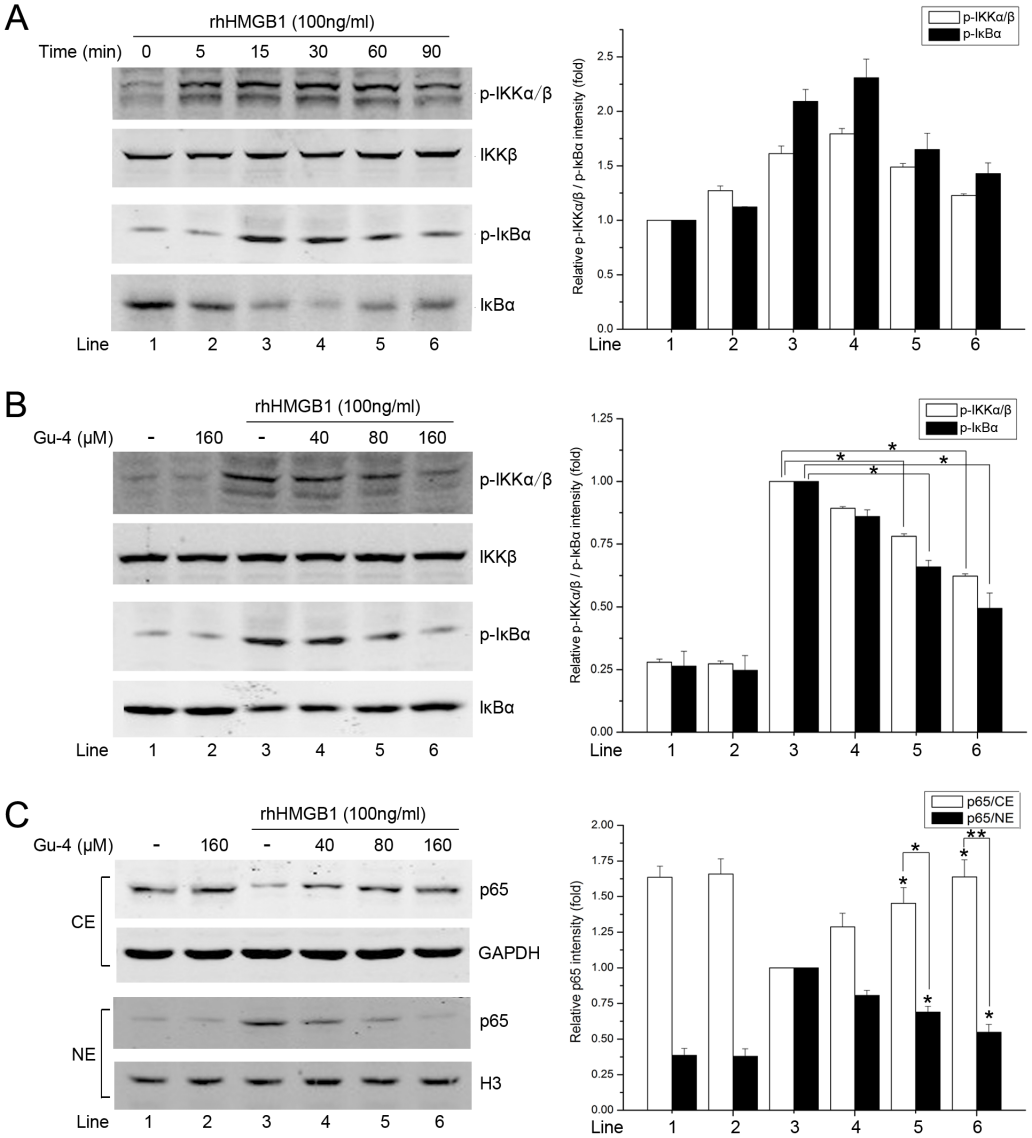
Gu-4 reduced rhHMGB1-elevated NF-κB activation. A: WB analysis of the activation of IKK and IκBα in THP-1 cells treated with rhHMGB1 (100 ng/ml) for 0, 5, 15, 30, 60 or 90 min, respectively. B: THP-1 cells were pretreated with or without Gu-4 (40, 80, 160 µM) for 1 h, followed by stimulating with rhHMGB1 (100 ng/ml) for 30 min. Phospho-IKKα/β and IκBα levels were determined by WB analysis. C: Detection of NF-κB p65 in the cytosolic and nuclear fractions from THP-1 cells which subjected to similar treatment as those in (B). CE:cytoplasmic extracts; NE: nuclear extracts. Data shown are representative results of triplicate experiments. *, *p*<0.05; **, *p*<0.01.

**Figure 8:**
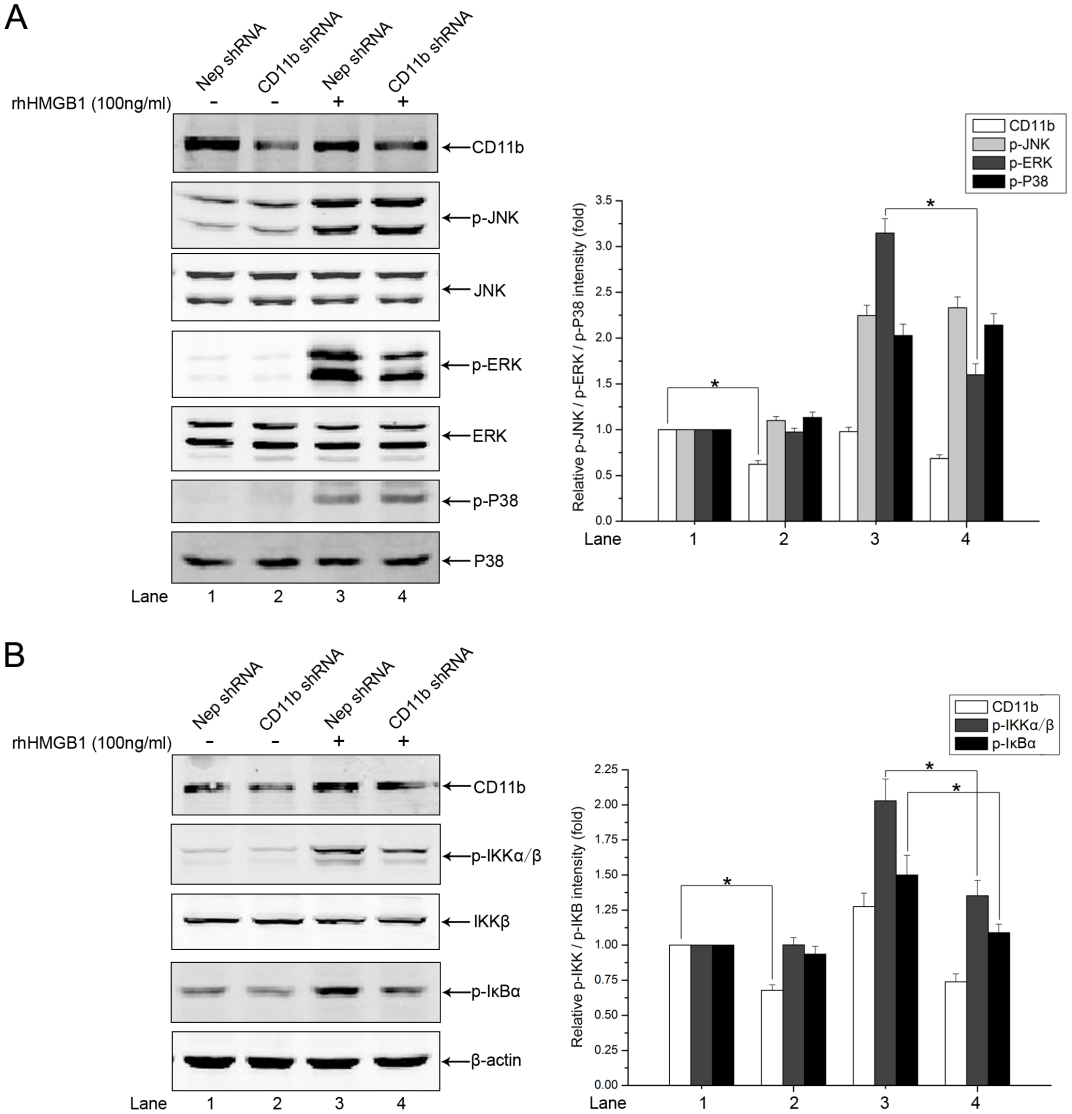
CD11b knockdown inhibited rhHMGB1-stimulated ERK and NF-κB activation. RAW264.7 cells were transfected with CD11b shRNA or negative control shRNA. 48(100 ng/ml) for 30 min. A: Western blot analysis of the phosphorylations of MAPKs including JNK54/46, ERK1/2 and p38. B: Phospho-IKKα/β and phosphor-IκBα levels were determined by western blot analysis. Nep: negative control plasmid. The experiments were performed in triplicate. *, *p*<0.05.

Pro-inflammatory responses caused by extracellular HMGB1 correlate closely with MAPKs and NF-κB pathways [9], [11], [14]. Hence, we further investigated the effects of Gu-4 on the HMGB1 triggered downstream signaling events. The MAPK family mainly includes JNK, ERK1/2 and p38 MAPK. Western blot analyses showed that rhHMGB1 stimulation evoked obvious phosphorylations of JNK, ERK, and p38, which were reached the maximum at 30 min after the stimulation and then gradually attenuated (Fig. 6A). Gu-4 treatment reduced the rhHMGB1-induced phosphorylation level of ERK1/2, but not of JNK and p38, with a dose-dependent manner (Fig. 6B). In addition, the effect of Gu-4 on rhHMGB1-induced NF-κB activation was also determined by Western blotting. As shown in Fig. 7A, rhHMGB1 induced the phosphorylations of IKKα/β and IκBα and the degradation of IκBα, which were all antagonized by Gu-4 (Fig. 7B). Next, we further verified that Gu-4 could inhibit NF-κB signaling by detecting the nuclear translocation of p65 subunit of NF-κB. Consistently, rhHMGB1 induced a major expression of p65 in the nuclear fraction, while Gu-4 reduced the nuclear expression of p65, indicating that Gu-4 inhibited the rhHMGB1-induced p65 nuclear translocation (Fig. 7C). To find out whether CD11b is involved in the HMGB1-mediated activation of ERK and NF-κB, we knocked down CD11b using specific shRNA targeting CD11b. As shown in Fig. 8, upon the stimulation of HMGB1, CD11b knockdown cells exhibited obviously attenuated phosphorylations of ERK and IKKα/β compared to that of negative control transfected cells. This result clearly implies that CD11b plays a role in HMGB1-mediated signaling cascades, which is consistent with the above observation that Gu-4 targeted CD11b to suppress HMGB1 pro-inflammatory activity.

## Discussion

In our previous studies, we have demonstrated that Gu-4 could selectively target adhesion molecule CD11b to confer its beneficial effects in endotoxemic mice and in collagen induced arthritis of rats [24], [36], but the detailed mechanisms underlying Gu-4 anti-inflammatory function remains elusive. We previously showed that Gu-4 treatment did not alter the serum level of pro-inflammatory cytokines, TNF-α or IL-10, in LPS-induced endotoxemic mice. Therefore, in this study, we investigated the relationship between Gu-4 and HMGB1, a critical late mediator in endotoxemia and sepsis, and we found that Gu-4 inhibited the active release and the pro-inflammatory activity of HMGB1.

During sepsis, HMGB1 could be rapidly and passively released upon cell death due to infection and also could be actively secreted by immune cells in response to pro-inflammatory cytokines and pathogen-associated molecular patterns (PAMPs) [6], [37]. In the CLP induced septic rats, compared to serum TNF-α level reaching its peak at 3 h, the peak level of serum HMGB1 did not occur until relatively late at about 12 h; interestingly, *in vivo* administration of Gu-4 successfully inhibited the elevation of HMGB1 in CLP rats, suggesting that Gu-4 might act as an inhibitor on HMGB1 release and that the serum HMGB1 might originate mainly from the active secretion of LPS- and/or cytokines-primed immune cells. In order to discriminate between the active secretion and passive release of HMGB1 from cells, we thus examined whether Gu-4 affects cytoplasm-nucleus transfer of HMGB1, a key step for HMGB1 active release during inflammation. In a murine cell model of inflammation induced by LPS, we found that Gu-4 prominently deterred the LPS-induced translocation of HMGB1 from nucleus to cytoplasm.

When exposed to PAMPs and endogenously derived inflammatory mediators such as TNF-α, IL-1 and IFN-γ, monocytes, macrophages and other immune cells can actively secret HMGB1 through cellular signaling transduction initiated by plasma membrane receptor interaction with extracellular products. Active release of HMGB1 requires posttranslational modification (PTM) of specific lysines within the nuclear localisation sequence of HMGB1 [38]. Although we did not determine the PTM of HMGB1 in response to LPS and we carried out experiments only in the setting of LPS stimulated macrophages, we provided a valuable clue that the CD11b inhibitor such as Gu-4 might be used as a therapeutic agent for the blockade of HMGB1 active release in sepsis. Further detailed investigations need to be done to decipher the intriguing role of CD11b-mediated signaling transduction in the secretion of HMGB1 in the context of sepsis.

HMGB1 secreted into extracellular milieu can act as chemokine or cytokine to activate innate immune cells to produce pro-inflammatory cytokines [39]. The receptors on cell surface engaged in HMGB1 interaction include RAGE, TLR4 and TLR2. Particularly, it has been proposed that the activated CD11b participates in the signaling events associated with HMGB-RAGE interaction [26], [40]. It is also known that whether HMGB1 acts as a cytokine or chemokine is closely related with the state of three redox-sensitive cysteine on it (C23, C45 and C106); HMGB1 with its all three cysteine in their reduced form functions as a chemokine, while HMGB1 with C23-C45 disulphide bond but leave C106 in its thiol form behaves like a cytokine [17]–[18], [41]. The recombinant HMGB1 protein which we used in *in vitro* experiments did evoke a significant cytokine production (e.g., TNF, IL-6, and IL-1β) from THP-1 cells, indicating that the commercial available rhHMGB1 possesses cytokine nature. Interestingly, we observed that Gu-4 inhibited cytokine production from rhHMGB1 challenged THP-1 cells in a dose-dependent manner. Meanwhile, we found that Gu-4 inhibited the exposure of CD11b active epitope primed by rhHMGB1 and attenuated the adhesion of rhHMGB1-stimulated THP-1 cells to HUVECs. These data strongly indicated that activated form of CD11b is important for the achievement of pro-inflammatory activity of HMGB1. Further Western blot analyses showed that Gu-4 suppressed the rhHMGB1 triggered phosphorylation of ERK1/2 and nuclear translocation of NF-κB p65 which was consistent with inhibiting IKKα/β phosphorylation and IκBα degradation. These observations implied that Gu-4 targeting CD11b inhibits ERK and NF-κB signalings, and in turn, suppresses HMGB1 activity in production of pro-inflammatory cytokines such as TNF-α. Consistent with this implication, in Gu-4 treated CLP sepsis model, the second wave of serum TNF-α level was significantly decreased (Fig. 1D). All our data indicated that Gu-4 possesses a dual-role in suppressing HMGB1release and blocking HMGB1 pro-inflammatory activity.

HMGB1 has been proven to be a successful therapeutic target for the treatment of sepsis. Some HMGB1 antagonists have been successfully tested in experimental models, such as EP, HMGB1antibody and HMGB1 A-box [42]–[43]. Besides, several recent reports have demonstrated that some HMGB1 inhibitors can also inhibit LPS-induced HMGB1 release through distinct mechanisms. For example, nicotine prevents the activation of NF-κB pathway and inhibits HMGB1 secretion through a mechanism dependent on the α7-nicotinic acetylcholine receptor (nAChR) [44]; EGCG, a green tea major component, inhibits the LPS-induced HMGB1 up-regulation and the extracellular release by stimulating LC3-II production and autophagosome formation [45]; tanshinone IIA sodium sulfonate (TSN-SS) inhibits the LPS-induced HMGB1 release by facilitating HMGB1 endocytic uptake [46]. Here, we provide some suggestive clues to use Gu-4 to intervene HMGB1 and the underlying mechanisms involve targeting CD11b I-domain by Gu-4.

In conclusion, we evaluated the therapeutic effects of Gu-4, a previously identified CD11b inhibitor, in a CLP sepsis model of rats, and explored the underlying mechanisms by performing a series of *in vitro* experiments. Our results demonstrated that Gu-4 significantly improved the survival of CLP animals, which could be attributed to: 1. Gu-4 significantly suppressed the LPS triggered cytoplasmic translocation and active release of HMGB1 in macrophages. 2. Gu-4 reduced HMGB1-stimulated production of pro-inflammatory cytokines from macrophages. 3. Gu-4 inhibited HMGB1-induced interaction between leukocytes and endothelial cells. 4. Gu-4 inhibited HMGB1-mediated ERK and NF-κB activation through targeting CD11b. All our data indicates that Gu-4 possesses a dual-role in suppressing HMGB1release and blocking HMGB1 pro-inflammatory activity.
